# The Implementation of Mindfulness-Based Programs in the Swedish Healthcare System—A Qualitative Study

**DOI:** 10.1177/21649561211058698

**Published:** 2021-11-26

**Authors:** Susanne Andermo, Rebecca Crane, Maria Niemi

**Affiliations:** 1 Department of Global Public Health, 27106 Karolinska Institutet, Stockholm, Sweden; 2 Division of Nursing, Department of Neurobiology, Care Sciences and Society, 27106 Karolinska Institutet, Stockholm, Sweden; 3 Centre for Mindfulness Research and Practice, School of Psychology, 1506 Bangor University, Bangor, UK; 4 Center for Social Sustainability, Department of Neurobiology, Care Sciences and Society, 27106 Karolinska Institutet, Stockholm, Sweden

**Keywords:** mindfulness-based programs, national guidelines, implementation, intervention integrity, fidelity

## Abstract

**Background:**

As the provision of Mindfulness-Based Programs (MBPs) in health care settings progresses, more research is needed to develop guidelines and structures for implementation in various contexts. This study is part of a larger project were MBP provision in Sweden is explored.

**Objective:**

The objective is to provide knowledge for the next steps of MBP implementation both in Sweden and internationally. The specific aim of the study is to explore how MBP teachers and other relevant stakeholders experience the implementation of MBP.

**Methods:**

Qualitative in-depth interviews were conducted with 15 MBP providers and 2 other stakeholders from a range of health care settings in Sweden.

**Results:**

The results, presented in 3 themes, provide insights into the factors that are crucial for facilitating or hindering MBP implementation; (1) MBP teachers and their training, including the importance of champion individuals and the benefit and shortcomings of various forms of MBP; (2) Patients and patient referrals, including patient characteristics and referral pathways; (3) Organizational prerequisites to successful implementation, highlighting the importance of financial factors and managers’ and colleagues’ knowledge and acceptance of MBP; and (4) the need for structural changes, including future recommendations on quality assessment and guidelines.

**Conclusion:**

This study highlights the need for national guidelines for MBP provision and teacher training pathways, as well as improved availability of teacher training. Also, the benefit of a stepped-care model of MBP provision is indicated by the findings. Finally, increasing awareness of MBPs among referrers, managers, and the public may enable successful implementation.

## Background

Mindfulness-Based Programs (MBPs) have in recent years gained a growing body of evidence for their use in clinical settings, primarily for the treatment and management of Common Mental Disorders. A number of countries have included MBPs in their national guidelines for depression treatment and relapse prevention. For example, the American Psychological Association, recommends Mindfulness-Based Cognitive Therapy (MBCT) for depression treatment and relapse prevention among the general adult population,^1^ and the Canadian Network for Mood and Anxiety Treatments, recommends MBCT as a first line treatment for maintenance treatment of major depressive disorder.^2^

Crane et al^3^ aimed to develop a consensus (of the lead MBP developers internationally) definition of the key essential elements that define all MBPs: they are (1) informed by theories and practices that draw from a confluence of contemplative traditions, science, and the major disciplines of medicine, psychology and education; (2) underpinned by a model of human experience which addresses the causes of human distress and the pathways to relieving it; (3) develop a new relationship with experience characterized by present moment focus, decentering and an approach orientation; (4) supports the development of qualities such as joy, compassion, wisdom, equanimity and greater attentional, emotional and behavioral self-regulation; and (5) engage participants in a sustained intensive training in mindfulness meditation practice, in an experiential, inquiry-based learning process and in exercises to understanding.” This definition has been adopted as an anchor point to support integrity in research and implementation of MBPs worldwide, including by the International Mindfulness Integrity Network^4^ which disseminates good practice for MBP teaching practice. The most widely researched and implemented MBPs are Mindfulness-Based Stress Reduction (MBSR)^5^ and MBCT.^6,7^

Researchers have detailed that these programs reduce illness and distress, and promote wellbeing and flourishing by improving metacognitive skills and self-regulation of attention and emotions.^8-10^ The psychological mechanisms associated with the clinical benefits of MBSR and MBCT include enhanced emotional regulation, higher self-compassion and metacognition, decreased rumination and worry, and decreased experiential avoidance.^11-13^

### Implementation of MBPs in Sweden

In response to growing evidence for MBPs they have been included in the depression treatment guidelines of the Swedish National Board of Health and Welfare in December 2017.^14^ The guidelines recommend MBCT for relapse prevention in depression, while stating that more research is needed for the use of MBCT or MBSR in treating mild-moderate depression or anxiety.^14^ However, there is a lack of systematized knowledge regarding the implementation of MBPs—including the types of programs being provided, the level of training among MBP teachers, and the extent to which programs are available—in the Swedish health care context. Furthermore, there is a lack of knowledge about what hinders and facilitates the uptake of MBPs, to inform how to best implement MBPs in the Swedish health care system. A recent qualitative study in Sweden, where MBP was provided for pregnant couples with increased risk for stress and depression, has provided some useful preliminary understanding regarding possible sources of MBP participant motivation and skepticism. While a majority of the participants experienced MBP as helpful in many ways, others found that the mindfulness practices did not suit them and their skepticism toward the practice clearly hindered them for experiencing MBCP as valuable.^15^ Implementation has been defined as the “act of putting a plan into action or of starting to use something in practice.”^16^ Implementation research is a broad field that addresses different aspects of implementation; including the processes of implementation, strategies that are needed for implementation as well as implementation outcomes.^17^

As the implementation of MBPs in health care settings is increasing globally, more research is needed to guide structures and guidelines for the implementation journeys in various local context. This study, therefore, provides an in-depth view of the implementation processes that have taken place in Sweden in order to illustrate some generalities and specificities of MBP implementation in the light the particular context of Swedish health care. The present study is thus part of a wider project which also includes a quantitative survey study, which aimed to map the provision of MBPs of in Swedish health care.(18) The aim of this study is to add depth and detail to the understanding gleaned in the linked survey study to better inform the next steps in the implementation journey both in Sweden and internationally. The specific aim of the present study is to explore how MBP teachers and other relevant stakeholders experience the implementation of MBP in the Swedish health care setting.

## Methods

### Design

The study is part of a larger project examining and exploring existing MBP provision in Swedish health care systems with qualitative and quantitative methods. The overall design of the project was inspired by a UK trial mapping the uptake of Mindfulness-Based Cognitive Therapy in the national health service,^19^ and was informed and structured by the Promoting Action on Research Implementation in Health Services (PARIHS) framework.^20^ In this study, a qualitative approach is used to gain an in-depth understanding of different stakeholders’ experiences of the process of MBP implementation. The participants in this study were identified through a survey used in the quantitative part of the project, in which existing MBP provision in Sweden was systematically mapped using a survey developed within the context of the UK study.(21)

### Data Collection

129 participants from 20 of the 21 regions in Sweden answered the survey and were asked if they were interested in participating in an interview. 39 participants from 15 regions in Sweden volunteered for a follow-up interview. All were MBP teachers, with various forms and levels of experience and education. Participants for the qualitative interviews were selected to include participants from (1) different regions, (2) with an equal distribution of males and female and (3) a variation of mindfulness teacher training background, and (4) a variety of health care professions. Based on this selection, 19 survey respondents were invited by e-mail to participate in an interview. A reminder was sent to those who did not respond approximately two weeks after the first invitation. Of those who were invited, 15 agreed to participate in the interview, two declined, and another two did not respond to the invitation or the reminder. In addition, all interview participants were asked to recommend a co-worker who could also give their view of MBP provision in the service. Two participants recommended co-workers. Two co-workers agreed to participate, two declined participation, and one did not respond.

A semi-structured interview guide was constructed inspired by the PARIHS framework, and by themes arising through the analysis of the survey results (see Table 1). The interview guide had open-ended questions to explore MBP teachers’ experiences of MBP implementation. The questions related to (1) the context for implementation of MBPs in their workplace (i.e., kinds of patients, referrals processes, and other characteristics of implementation), (2) the process of implementation, including the development of MBP over time and facilitators and barriers for development and (3) the evidence-base, including teacher training of MBP providers and quality assessment of MBP delivery. The question guide for the co-workers contained similar questions adopted to explore their perspectives.

**Table 1.** Question guide for mindfulness teachers.

The context for implementation
• Can you tell me how it came to be that you started with mindfulness-based practice (MBP)?
• Can you tell us more about MBP within your workplace?
• Who’s taking the courses? (staff, patients)
• How do people come to the courses? (self-reporting, referred, how does it work)
The process of implementation
• What support do you think you need as an MBP teacher?
• Looking back, how has the MBP developed over time?
• Who (people) have been involved in the development of MBP at your workplace?
• How do you feel that the MBP have been received by others at your workplace?
• Are there any specific factors that have hindered development of MBP, can you describe these?
• Are there any specific factors that have promoted development of MBP, can you describe these?
• What would need to be adapted or changed to make it easier to work with MBP in your workplace?
• What policy decisions would be important for developing MBP?
The evidence base
• What thoughts do you have about MBP teachers’ needs for education and training?
• What are your thoughts and/or experiences about quality assurance of MBP?
• Are you conducting any evaluation of your MBP? If so, can you describe how?

Abbreviation: MBP: mindfulness-based programs.

All interviews were conducted by phone, due to the various geographical locations of the participants. The participants were informed about the study, first by e-mail, and then again by phone before the interview began, (see below for details on ethical issues). “The interviews with the MBP teachers lasted between 40 and 71 minutes and those with the co-workers and managers lasted between 20 and 27 minutes”…The interviewer aimed to be sensitive to the participants’ narrative, and asked follow-up questions to encourage the participants to elucidate further on issues of interest, in line with the interview guide. All interviews were conducted in Swedish, audio-recorded and transcribed verbatim by a professional typist. Quotes that are presented in this article were translated to English.

### Data Analysis

The data were analyzed using content analysis with a manifest approach.^22,23^ First, the transcripts were read several times by two of the authors (SA and MN) to get an initial understanding of the data. Thereafter, meaning units were abstracted into condensed meaning units and codes by the two authors. After the analysis of the first two interviews, the results were discussed with a focus on the differences and similarities to the interpretations. There was a high agreement in the analysis, including how meaning units were identified, condensed and abstracted into codes. The material was then divided into two parts and the two researchers performed the condensation and coding of half the material each.

After this, the analysis was discussed and codes with a similar content were grouped into themes and sub-themes. In this process, both researchers were able to cross-check each other’s interpretations and discuss and adjust the preformed coding. Themes, sub-themes, and a selection of quotes were translated to English. Drafts of the results were initially written in both Swedish and English by SA and MN, depending on their preferences. The Swedish parts of the results, and specifically the quotes were translated and edited mainly by MN, who is native to both languages. In addition, RC checked the quote translations in English, as she is a native English-speaker. The translations were discussed between SA and MN in order to maintain the initial meaning in the text.^24^ The emerging results were then discussed among all three researchers (SA, RC, and MN).

The researcher (SA), who conducted the interviews, has long-standing experience of qualitative methods, and asked open questions to allow for the participants to describe their experiences in their own words. SA has training in Public Health, Anthropology, and Caring Science, an interest in MBPs and experience in practicing mindfulness but she is not an MBP teacher. MN has long-standing experience of qualitative health care research. Two of the authors (MN and RC) are both experienced researchers in the field of MBPs. MN and RC are also qualified MBSR teachers and RC is a senior MBP teacher trainer.

### Ethical Considerations

The study was approved by the Regional Ethical Review Board in Stockholm (Approval number 2019-02952) and follows the ethical principles of the Declaration of Helsinki 1964. All participants received verbal and written information about the study and gave written informed consent. All participants were informed about the study, including how data were handled and stored as well as that their participation was voluntary*.*

## Results

### Participant and Service Characteristics

The study included 17 participants, of whom 15 were MBP teachers, one was a co-worker and one was a manager. The participants’ mean age was 51 years, range 31–63 years. They worked in various health care settings including: primary care, hospitals, private practice (with or without funding from publicly funded healthcare), and specialist care units (such as psychiatry, rehabilitation centers, a midwife clinic, ears, nose, and throat clinic, and a specialist care unit for brain damage). Three of the participants were nurses, and there were two each of the psychologists, social workers, midwives, and general therapists. The others were a physiotherapist, an occupational therapist, a curator, an educator, a social educator, and a physician.

The MBP teachers experience of mindfulness practice varied greatly, some had practiced for decades and others had just begun practicing in parallel with their MBP teacher training. Eleven of the teachers had training in the Swedish-specific Here and Now program^25^ and four were trained in MBSR and/or MBCT (Appendix 1). The manager and co-worker did not have a teacher training. Of the teachers, seven were currently delivering MBP groups in health care settings, five were not currently delivering due to different factors, and three delivered either a modified program or integrated elements of MBPs into individual patient sessions. Of those that had MBP groups, the majority were trained in the Here and Now program, and one was trained in MBSR (Table 2).

**Table 2.** Participant characteristics.

Participants, n:17	
MBP teachers (n)	15
Managers and co-workers (n)	2
Age, mean in years (range)	53 (31-63)
Female, n (%)	14 (82)
Male, n (%)	3 (18)
Teacher training in	
MBSR and/or MBCT, n (%)	11 (73)
Here and Now, n (%)	4 (27)

Abbreviations: MBP, mindfulness-based programs; MBCT, mindfulness-based cognitive therapy; MBSR, mindfulness-based stress reduction.

Four themes and nine sub-themes emerged in the analysis (see Table 3).

**Table 3.** Themes and Sub-Themes.

Themes	MBP teachers and their training	Patients and patient referral pathways	Organizational prerequisites to successful implementation	The need for structural changes
Sub-themes	A champion individual and the right conditions are key to success	A variety of patients in different settings	The organizational and financial prerequisites for group activities	Research, evaluation, and clear boundaries for MBP are essential forms of quality assurance
	The importance of MBP teacher training	Developing a collaboration with co-workers to enhance patient referral	The importance of managers’ and colleagues’ knowledge and acceptance	Structures and guidelines are lacking and needed
	Benefits and shortcomings of various forms of MBP			

## MBP Teachers and Their Training

### A Champion Individual and the Right Conditions Are Key to Success

The reality that successful implementation relies on the presence of a key person, who found themselves in the right conditions to drive the change process, was consistently expressed by the teachers. The teachers described a strong personal drive to implement MBPs in their respective workplaces. They have worked hard to convince their colleagues and leadership of the importance of implementing MBPs. A few teachers described that colleagues before them,, that were described as enthusiastic champions had initiated the implementation of MBPs in their workplace. These teachers also emphasized how they themselves committed deeply to the continuing the work to implement MBPs, following in their colleagues’ foot-steps.

Even though these teachers had a strong motivation, many of them were alone in delivering MBPs within their working context and this could lead to an excessive workload. One MBP teacher also described that there are two MBP teachers in her workplace, but that the management does not allow them both to deliver MBP:

*“When I was employed by the region… I said that I would like to start teaching MBSR groups ….. But they* [management] *believe that one teacher is all that is needed. There are* [up to] *24 participants each half year and I know that she always has a waiting list. So it is the management that said no.” *(Participant 8)

### The Importance of MBP Teacher Training

The teachers emphasized the importance of the quality of MBP teacher training. Having a formal teacher training was perceived as a basic condition for success in MBP implementation. However, for those who did not have a teacher training prior to starting their employment, the opportunity to attend training was an important starting point. Some had the training paid for and could attend during working hours, while others had to pay for themselves and attend the training in their spare time. In some cases, MBP teaching competence was a criterion for recruitment to the role:

*“I was employed here […] at the end of 2017, and already …at the job interview, they saw that I had competence in teaching mindfulness and that this was something patients had been asking for, … they currently did not have anyone skilled to deliver. So I started MBP implementation from scratch here when I started working.” *(Participant 7)

### Benefits and Shortcomings of Various Forms of MBP

The main programs for which teacher training is available in Sweden are the Here & Now program, and MBSR. Teachers had mainly chosen the teacher training that was most readily available in their region, or that they knew something about. The visibility of the Here and Now teacher trainer in popular Swedish media have increased its credibility for patients, and therefore made it the obvious choice for some to train in.

*“I often take Ola Schenströms book with me when I teach groups, and I mention his mindfulness center sometimes, because it gives a clear anchoring into something that people recognize. I think that this is important. That this isn’t something wishy-washy, but something that people know about, that they recognize his name and they’ve seen Ola on TV and have read his book and so on.”* (Participant 4)

The Here & Now program and MBSR differ in some respects, and some teachers weighed the benefits and shortcomings of these 2 programs against each other. Some, for example, reasoned that the teacher training pathway for MBSR is too long, and that health care workers thus have a hard time taking all the stages of training. Others also thought that the length of home practices in MBSR are not feasible for participants and thus preferred the shorter home practices assigned in the Here & Now program. Conversely, some others thought that the teacher training for Here & Now was too short and fell short of the depth offered by MBSR program and teacher training. In general, many emphasized the importance of personally experiencing depth of engagement with mindfulness practices, and that this was something that most in the health care services had very little knowledge about.

*“There’s a real lack of understanding in health care about [mindfulness] it’s not like you can just do a bit of mindfulness just like that. You are taking care of people who are ill and people who are not feeling well … one should understand that and know what can be brought up by the [meditation] practice and move slowly and carefully through it. And for that, you need a lot of competence. It’s not just about taking a short course and thinking you will just guide a meditation…the tough part with the MBSR program is holding these dialogues around these central themes that are difficult things to handle, and moving through it slowly with the group you have.”* (Participant 17)

## Patients and Patient Referral Pathways

### A Variety of Patients in Different Settings

The MBP teachers worked in different settings and had patients with a large variety of diagnoses and backgrounds participating at their MBP courses. Most commonly, patients suffered from mental health problems such as exhaustion, stress, anxiety, and depression.

*“It’s very mixed and very fun, mostly I work with stress-related ill-health; everything ranging from burnout to stress symptoms that people experience for various reasons, difficulties in handling life as it is. But I also have quite a few patients who have depression, anxiety, sleep problems, and some even have more severe problems. But worry, stress and anxiety I would say are the most common symptoms, and depression.”* (Participant 4)

However, there were also other patient groups including patients with tinnitus, endometriosis, brain damage, sexual health problems.

### Developing a Collaboration With Co-workers to Enhance Patient Referral

The MBP teachers emphasized the importance of good collaboration with co-workers to enhance patient referrals to MBP sessions. Patients most commonly came to MBPs through referrals from other healthcare providers, on their own initiative or through recommendation from MBP teachers. Some teachers worked actively to get more participants in their groups by putting up posters and informing colleagues. Often, teachers described that it had been easy to recruit patients:

*“People are more interested nowadays, actually they call and sign up themselves. And also, this is a very small municipality compared to many other places, so the positive reputation spread here: “I have a friend who has done this course and I myself have problems, and she tells me this would be good for me. Do you think there’s a possibility for me to get this too?” So there’s a lot of positive response.” *(Participant 10)

Most of the teachers experienced that there were a couple of colleagues who referred the main share of patients, while other colleagues referred only a few or no patients. This was thought to depend on their colleagues’ interest in and understanding of MBPs. A strategy that some teachers used to get more patients on their courses was to develop collaborations with colleagues and increase their awareness of the benefits and applications of MBP.

*“In the beginning they were mainly my own patients, but then the word spread and I spoke to people at the clinic, made them aware that this exists. So then there were doctors who referred patients to the courses. And then other caregivers had patients who they sent to the groups, so it became a complement to other psychological care.” *(Participant 13)

Patient satisfaction with the MBP courses and their experience of positive effects were seen as factors that motivated the teachers and promoted implementation. The patients' positive response was described as an essential factor that contributed to colleagues being made aware of the benefits of MBP implementation.

## Organizational Prerequisites to Successful Implementation

### The Organizational and Financial Prerequisites for Group Activities

The MBP teachers described a variation in the availability of organizational and financial prerequisites for conducting group activities. For some, it was possible to have groups as part of their defined job role. These teachers justified group activities from a cost perspective when they can meet several patients at the same time:

Mindfulness-based programs teacher: “During the 8-week program the first session I have is 3 ½ hours long, and then the following 7 times we meet for 2 ½ hours, so they’re quite hefty sessions.

Interviewer: *Yes indeed, and so you get reimbursement for those hours then?*

Mindfulness based programs teacher*: Yes, well we get reimbursed when we have groups, we* [the health care company] *get 300 kr* [36 USD] *per group participant. That’s different when I meet patients for individual therapy, (…), so we end up on the plus side financially. So, I have increased the economic gains here for the company and I’ve been able to shorten the patient waiting lists and been able to offer methods that are alternatives to those we already offer, so it’s a win-win concept really.” *(Participant 4)

Having funding to conduct MBP classes was a crucial factor. Although some teachers described that they were able to give MBP classes without any budget concerns, most teachers described that the lack of availability of funding negatively affected MBP activities. Activities were sometimes financed though specific, non-permanent efforts, such as research and development projects, which led to uncertainty and a lack of long-term sustainability. The availability of funding as well as the ways in which group activities were funded could also change over time:

*“In the beginning … until 2016 or -17, group interventions were included in the selection of psychotherapy interventions that we have available, but that changed from 2017 and then group interventions weren’t included in the selection anymore.”* (Participant 2)

Others also described that no specific funding was available for MBP delivery. For example, the organization’s protocols did not allow MBP teachers to register when patients participate in group meetings because the meetings were often longer than standard individual sessions. In particular, it was a challenge to organize 2.5 hour long group sessions, as these could not be charged-for within the existing funding system. However, other MBP teachers were able to find loopholes around these challenges:

*“They only participate in the group activities, and that means that we can include many more patients in a meeting than is usually possible. This means that we gain more time in our schedules for additional patients. And I think that we need to think a little bit about this […] there’s great pressure on individual psychotherapy services now and at the same time there’s this funding available, so we need to plan this better. We need to help more people a little bit instead [of helping only a few in depth].”* (Participant 16)

However, for others, these organizational bureaucratic challenges caused problems, and drove reductions in the length of sessions. Some teachers reported that they have resorted to only implementing mindfulness practices during individual visits because of these factors. Adequate premises for organizing group activities were something that some, but not all, had access to.

### The Importance of Managers’ and Colleagues’ Knowledge and Acceptance

Positive support from managers and colleagues, such as knowledge and understanding, is described as an important factor for the implementation. Mindfulness-based programs teachers who described their manager as supportive emphasized how important this has been as a factor for success. Other factors were to get freedom as a MBP teacher to develop the MBP programs as well as to get the necessary prerequisites for it. Some teachers describe that they have felt affirmed by the confidence the manager has placed in their activities. Another MBP teacher described how an initially supportive manager changed in approach and suddenly decided that the MBP activities could not continue. The MBP teacher emphasized the importance of establishing contacts with varying levels of leadership:

*“So the boss plays a central role in this, you need to establish [MBP understanding] with those above you, you need to have them on board, and that’s where I didn’t do enough. I should have worked much more strategically to establish these activities with them, and to invite them in.” *(Participant 1)

Mindfulness-based programs teachers also described how they have worked actively to increase the understanding of MBP among their managers and colleagues. Some offered taster sessions and opened up MBP classes for colleagues, which has led to an increased understanding and knowledge about MBPs in their work context:

*“I’ve been out and about and delivered talks (…), I’ve offered a couple of lectures and we’ve had an open house (drop-in session) here at the health care center where I’ve also delivered lectures and taster sessions in mindfulness.* (Participant 10)

Some MBP teachers experience skepticism from colleagues about their MBP work. Some described that colleagues laugh behind their backs and disparage and devalue their work. One teacher described that there is some fear and a resistance to mindfulness in her surroundings:

*“One thing I wanted to say is that one notices really how hard it is to implement [mindfulness] you know, there is this general fear that it’s “New Age”. We live in a society where we follow facts and figures and we’re happy with that. We’re a statistics society one could say a materialist society. And people don’t take in new things and don’t think that the spirit also needs to be taken care of. And yeah, there’s so much of that in our society, and the doctors are not receptive, and many don’t have knowledge about taking care of their patients from a psychological perspective.” *(Participant 9)

## The Need for Structural Changes

### Research, Evaluation, and Clear Boundaries for MBP Are Essential Forms of Quality Assurance

Many teachers emphasized the importance of research—that which has already been conducted as well as the need for further studies on particular programs and patient groups – to underpin quality assurance of MBP delivery. In addition, the importance of continuous evaluation of participant outcomes was emphasized by some, in order to ensure high-quality delivery. Such evaluations might include self-assessment questionnaires as well as reports of patient feedback, and several teachers do indeed utilize these forms of evaluation. Some conducted their own research on the MBP delivery and highlighted this as an implementation facilitator. Indeed, these various forms of participant outcome evaluation that could be used to communicate MBP benefits to health care management were highlighted as strong enabling factors for implementation.

Some teachers voiced concerns about the lack of clear guidelines for patient referral to MBPs. They highlighted that referrers as well as MBP teachers need access to inclusion- and exclusion criteria for MBP that are guided by an awareness of possible adverse effects for example for those with trauma backgrounds. Before patients joined MBP programs, many teachers assessed their patients’ health status, motivation, and expectations to emphasize that patients need to be prepared for the engagement that is involved in taking the course. Some teachers also clarified that they did not include all patients:

*“I only include those with mild or moderate severity, not severe or high levels of anxiety or depression.” *(Participant 9)

To ensure quality, a recommendation to increasingly take these aspects into account was voiced by some:

*“I think generally within heath care there is a romanticized view of mindfulness as if it’s something totally benign that doesn’t cause any harm to individuals. For me, that’s a problematic attitude… We, as teachers need to for example be attentive if a participant becomes hyperactivated.... We need to know how to help and guide the group to support them self-regulate […] and also if someone becomes a bit slumped and dissociated….. we need to have a check on hyper- and hypo-activation so they don’t become dysregulated.”* (Participant 3)

On the other hand, another teacher was of the opinion that the use of specific exclusion criteria would be unfortunate, as many patients who may have benefited greatly from MBP may thus be excluded due to unnecessary precaution:

*“I’m really allergic toward this business of setting up of too many criteria and that they start to do this with mindfulness as well, as they do in health care in general. That ”you are excluded, you are included”, I don’t like it. Rather, I would say, “come and try it out“.”* (Participant 4)

Another delineation that was described as important for quality assurance was that teachers should be clear about the boundaries between secular mindfulness and mindfulness in Buddhist contexts or New Age philosophies:

*“I notice that there’s this thing, that people who do these things sometimes are really interested in going on retreats, and that there’s this whole “hallelujah” attitude around it. … they have this deep personal engagement … they are fascinated by the Buddhist perspectives, […] for me this stuff is not so important because if I were to do that, then my patients would wonder “are you Buddhist?”.. “*(Participant 2)

### Structures and Guidelines Are Lacking and Needed

Many teachers voiced frustration about the lack of clear frameworks for good practice for MBP teachers in Sweden. This was an implementation hindering factors as it resulted in lack of knowledge about MBPs within the organizations in which they worked relating to teacher training processes, and a lack of understanding and respect for the level of competence needed by MBP providers. An MBP teacher said: *”It seems like at my work place, it’s a bit like anyone can do it as long as you’ve read about it a bit.” (Participant 17)* This lack of guidelines also applied to the commissioners at a regional level:

*“I think a dream scenario would be that they, the commissioners, at the regional level, would know the difference between different levels of teacher training. That they would set a minimum level that is required, and that they would decide that now it’s good to send people for training in […]MBSR-groups, or that they […] would have another, say shorter training or group activity where you don’t do the 8 weeks, but that they would know the difference. ‘Because in general, people don’t know.’”* (Participant 8)

Some teachers considered that the competence levels of MBP teachers should be clearly reflected in the levels of compensation that they get for their work:

*“I think for example that it should generate a higher level of compensation if I am a certified teacher. I have reached a certain level for teaching MBSR and follow the curriculum which includes 2.5 hour sessions and it would be fair if that gave me a higher level of compensation than if I had say participated at a 5 day teacher training in mindfulness or if I teach a course that I myself have made up.”* (Participant 3)

## Discussion

This study included 15 providers and two managers of MBP services from a broad range of health care settings and regions in Sweden. The findings have shed light on the various ways in which MBPs are implemented in services, and how and why they are being modified to fit local contexts. This study provides insights into the implementation process and factors that are crucial for facilitating or hindering MBP implementation in a variety of Swedish health care services and therefore add nuance and depth to the quantitative study findings.(18) The themes that emerge though the qualitative analysis included the following: (1) MBP teachers and their training; (2) patients and patient referrals; (3) organizational prerequisites to successful implementation; and (4) the need for structural changes.

In relation to the PARIHS framework, the implementation process can be understood as a dynamic process of context, evidence, and facilitation.^20^ In terms of evidence, several forms of evidence were considered and used by the MPB teachers, such as research, patients experiences, and the teachers own professional experiences. Some teachers wished for clearer governance for MBP provision in health care, for example, in the form of national good practice guidelines such as are available in other countries—as detailed in the introduction. Mindfulness-Based Cognitive Therapy is mentioned in the Swedish guidelines for depression and anxiety healthcare which provides impetus for national roll out. Currently, however, there is a lack of supporting structures such as quality standards for teachers to support the quality of implementation efforts. By contrast, with regard to Cognitive Behavioral Therapy and psychodynamic therapy, the Swedish National Board of Health and Welfare provides information about the scope and structure of treatment, and licensed practitioner training institutions are listed.^26,27^ National guidelines for MBPs could be inspired by those that are in place in the UK. There, MBCT is listed as a mandated intervention for anxiety disorder and depression treatment within the Improving Access to Psychological Therapies Expansion Programme of the National Health Service.^28^ This inclusion is accompanied by a manual detailing the national MBCT teacher training curriculum and pathway.^29^ Indeed, such clearly stated guidelines would clarify which form of MBP should be implemented for which context and population, with appropriate training pathways tailored to the Swedish context. This would provide guidance for those health workers wishing to train as an MBP teacher, so that practitioners would need to stop “guessing” what the best training pathway is, as some of our participants indicate is necessary in the current situation. Indeed, as discussed by Dimidjian and Segal,^7^ “the thorny question of clinician training” (p. 605) has not yet been resolved by research, and recommendations differ widely across MBPs with respect to formal training for teachers.^30^ Empirical investigation of these issues is needed in order to develop clearer, evidence-based guidelines in this respect.

Another aspect that several our participants’ emphasized was the difficulty of funding teacher training. Indeed, in Sweden, other psychological therapies such as Cognitive Behavioral Therapy and Psychodynamic Therapy, that are mentioned in the national guidelines for depression and anxiety guidelines,^14^ are taught at universities and thus financed by the government. As of yet, MBP teacher training is provided by private practitioners, and participation is either paid out-of-pocket or by some limited regional initiatives, as witnessed by our participants. By contrast, in countries such as the UK where MBP delivery has become more formalized in national guidance, training is provided by mainstream institutions such as universities, or by training providers within the health service. The availability of all stages of teacher training and certification in Sweden, and preferably available in local regions, are important. Teacher credibility to participants was perceived as important and could be improved by increasing public knowledge about the program through dissemination by public figures in popular media. Another possible means of increasing public awareness is the formation of national associations for MBPs, along the lines of the British Association for Mindfulness-Based Approaches^31^ and the European Association of Mindfulness-Based Approaches.^32^ Such associations can support the development of national good practice guidelines, and be a vehicle for communication with the public and decision-makers.

Another important aspect of note, mentioned by some of participants in our study, is the current development of research into possible harms of MBP for some patient groups.^33,34^ Indeed, some of our participants mentioned that they utilized various screening procedures for including participants to their programs. Some participants emphasized the importance of such procedures, referring to emerging evidence of possible adverse effects of MBPs for some groups of patients.^35^ Others had a more open approach to including participants, where they did not apply any inclusion/exclusion criteria. This is yet another area that national guidance is required.

An important theme that was raised was the importance of delivering MBPs in ways that are clearly secular, without connotations to Buddhism, New Age, or other associated, cultural or religious practices. Indeed, Crane delineates the ways in which MBPs often have been caught in a cross current of divergent criticisms when attempting to maintain their key tenet and ethic of teaching a form of mindfulness that is not Buddhist and thus universally accessible. Mindfulness-Based Programs have been critiqued from two sides as either too Buddhist to be provided within tax-paying national health services, while proponents from the Buddhist perspective have regarded MBPs as insufficiently Buddhist, thus risking watering down and losing the important essence and ethical underpinnings of mindfulness practice.^36^ According to the World Values Survey,^37^ Sweden is at the furthest extreme in being a society characterized by secular values, and this may imply that concerns regarding clear delineations from religious practices are of particular concern in the Swedish setting, which one of our participants referred to as a “statistics society”. In this sense, the implementation of MBP in the Swedish health care was adjusted to contextual factors. Contextual factors, such as culture, leadership and evaluation have been highlighted as important to promote successful implementation. In the PARHIS framework, learning organizations with transformational leaders are emphasized to have a key role in implementation.

It is clear that a number of our findings from this qualitative study parallel those in the UK MBP implementation research.^19^ These include the importance of champion individuals driving implementation, and their engagement as facilitators for MBP in a range of proactive activities such as forming networks within and beyond the organization, and catalyzing interest through delivering taster sessions and talks. Also, our findings illustrate various ways in which “stepped care” of MBP implementation in health care, as suggested by Demarzo and co-workers,^38^ and as suggested in our other study.(18) Indeed, some interview participants were proponents of providing shorter versions of MBPs, due to feasibility issues and this may indeed be adequate in cases of milder health conditions.

## Methodological Discussion

This study has strengths and limitations. Strengths included that the participants represented a broad range of MBP providers from a variety of health care settings and region in Sweden, thus providing a multifaceted perspective of MBP implementation; and that providers from a variety of MBP programs were represented, thus providing perspectives on the possible benefits and shortcomings of the variety of different program models. This allowed us to gain understandings that could seem contradictory at first sight: that is, some of our teachers were proponents of shorter programs due to concerns of accessibility, while others were proponents of full MBP programs due to concerns of fidelity. However, these views need not necessarily be contradictory, but may also complement each other to inform the implementation efforts of MBP in Sweden in the future, by for example as suggested above, developing a stepped-care implementation model. A limitation of the study was, however, that most teachers were trained in the same training center.

All interviews provided rich material for analysis. The length of the interviews with the co-workers and the managers was shorter than those with MBP teachers, mainly due to time limits in their schedule. A methodological strength of the study was that two researchers conducted the qualitative analyses, thus increasing reliability. The two researchers had complementary perspective in the analysis (one as an MBSR provider and one not) which enabled a beneficial triangulation of various perspectives in the analysis and interpretation of results. Furthermore, during data collection, the interviewer was unfamiliar with some aspects of MBPs that the participants brought up, and handled this by asking follow-up questions to clarify what the participants meant. This approach contributed to providing a rich material.

A weakness of the study was the lack of inclusion of referrers and managers who would have provided additional beneficial insights. Attempts to recruit this group were unsuccessful. Future studies in Sweden would preferably also address the view of other health professionals and managers in regard to MBP implementation. Financial and time restrictions constrained us from extending the study recruitment further, and some of those who were approached declined participation due to time constraints inflicted by the Covid-19 pandemic and its impact on health services. Therefore, our study does not provide a more complete “case study” perspective as is presented in the UK MBCT implementation trial.^19^

## Conclusion

Our aim in this study was to hear various perspectives on MBP implementation, including those of providers, managers, and referrers to programs. This shed light on the important factors hindering or facilitating implementation. In summary, participants clearly voiced the need for national guidelines for MBP provision and teacher training pathways, as well as improved availability of teacher training. Also, the various and sometimes contradictory participant perspectives can be added up to indicate the benefit of a stepped-care model of MBP provision. In the light of various factors that limit the possibilities for full program implementation in a number of health care settings, stepped-care models could imply full programs being delivered for those with more severe conditions, where adequate screening procedures should be put in place in order to ensure safe provision of MBP. On the other hand, shorter or in other ways less intensive programs may be of benefit for those with less severe conditions. Increasing awareness of MBPs among referrers, managers as well as the public was voiced as an important concern by our participants and may enable more targeted implementation for patient groups for whom MBPs are most suitable. A national Association for Mindfulness-Based Approaches could provide a vehicle for communication with the public and decision-makers.

## Appendix 1

### Box 1: Teacher training pathways in Sweden

Some MBP teachers in Sweden may have undertaken their teacher training abroad, for example, at Brown or Bangor University. However, the majority will have trained in Sweden. There are two main providers of MBP teacher training in Sweden: Center for Mindfulness Sweden, providing teacher training in MBSR, and Mindfulnesscenter, providing teacher training in an MBP that is a modified version of MBSR, called the Here & Now program. Center for Mindfulness Sweden The teacher training at Center for Mindfulness Sweden trains in MBSR and MBCT, and follows the principles and standards for good practice that have been put forth by the International Integrity Network in Mindfulness-Based Programs.^1^ Teacher training includes participation in the following steps, after the completion of which the trainee may apply for teacher certification:

1. An 8-week MBSR or MBCT program
2. A 5–10 day teacher-led mindfulness retreat
3. An 9 day teacher training
4. Another 5–10 day teacher-led mindfulness retreat
5. An advanced teacher training, 8 days
6. Two further 5–10 day teacher-led mindfulness retreats
7. A 5 day MBCT add-on program, for those wishing to teach MBCT
8. Group supervision online, 10 times 60 minutes
9. Individual supervision, 10 times, 50 minutes
10. Teaching at least 8 MBSR programs

Mindfulnesscenter Mindfulnesscenter provides teacher training in the Here & Now program, which is inspired by the MBSR and MBCT programs and developed since 2004 by Ola Schenström, MD. The program involves 6 weekly 2 hour sessions and 20 minutes of daily home practice. The program has been assessed in a randomized controlled trial, with a non-inferiority design comparing the Here & Now program to Cognitive Behavioral Therapy (CBT) among 196 primary care patients with depressive, anxiety, or stress and adjustment disorders. The results showed that Here & Now performed equally well as CBT at post-intervention.^2^ The teacher training for Here & Now involves participation in the following steps:

1. A 6 week Here & Now program
2. A 6 day stage 1 training
3. Participation in an online MBCT program
4. A 5 day teacher-led mindfulness retreat
5. A 6 day stage 2 training
